# How T-lymphoblastic leukemia can be classified based on genetics using standard diagnostic techniques enhanced by whole genome sequencing

**DOI:** 10.1038/s41375-022-01743-6

**Published:** 2022-11-05

**Authors:** Janine Müller, Wencke Walter, Claudia Haferlach, Heiko Müller, Irene Fuhrmann, Martha-Lena Müller, Henning Ruge, Manja Meggendorfer, Wolfgang Kern, Torsten Haferlach, Anna Stengel

**Affiliations:** MLL Munich Leukemia Laboratory, Max-Lebsche-Platz 31, 81377 Munich, Germany

**Keywords:** Acute lymphocytic leukaemia, Cancer genetics

## To the Editor:

With the introduction of the 5th edition of the WHO classification, the number of genetically defined entities in myeloid neoplasms and BCP-ALL has increased considerably [1, 2]. However, no genetically defined entity has been introduced in T-lymphoblastic leukemia (T-ALL), as genetic group assignment remains complex and the reproducibility between studies varies. In the International Consensus Classification (ICC) eight subgroups (*HOXA*-dysregulated-, *SPI1-*, *TLX1-*, *TLX3*-, *NKX2*-, *TAL1-2*-, *LMO1-2*-rearranged T-ALLs and T-ALLs with rearrangements with other helix-loop-helix family members like *LYL1* or *OLIG2/BHLHB1)* have been proposed as provisional entities, due to lack of consensus how to define different subtypes [3]. However, for a first step towards personalized medicine a distinct classification based on biomarkers assessable by routine diagnostic methods is essential. Thus, we analyzed 131 T-ALL sent to MLL Munich Leukemia Laboratory between 05/2008 and 12/2020 by chromosome banding analysis (CBA) ± fluorescence in situ hybridization (FISH) on interphase nuclei. Additionally, WGS (100×, 2 × 151bp) and WTS (50 Mio reads, 2 × 101 bp) were performed on a NovaSeq(ILMN). Variants were called with Strelka2, Manta and GATK using a tumor w/o normal pipeline, fusions with Arriba, STAR-Fusion and Manta. T-cell receptor (TCR) rearrangement analysis was based on WTS data (Supplementary material and Supplementary Table 1). All patients had given written informed consent to the use of genetic and clinical data according to the Declaration of Helsinki. The study was approved by the internal institutional review board of MLL.

**Fig. 1:**
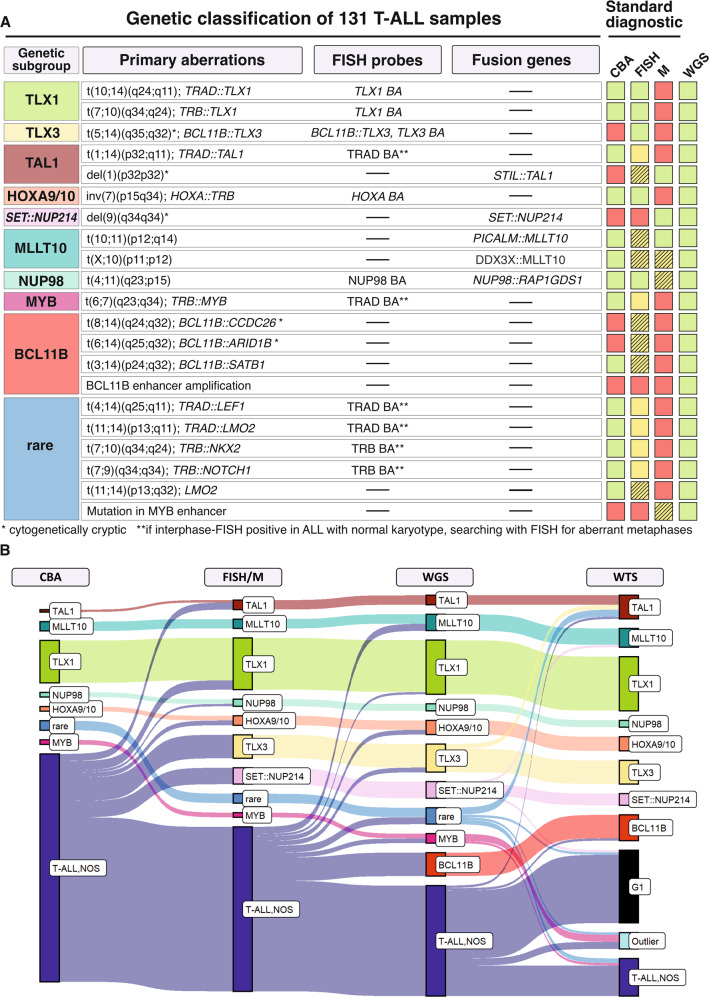
Definition and detection of T-ALL subtypes. **A** In conjunction with cytogenetics, molecular genetics and WGS, the cohort was classified into 9 distinct subtypes based on their primary genetic event. While translocations can be detected with CBA or commercially available FISH probes, gene fusions are detected by molecular genetics. The drawing on the right shows which method is suitable for detecting the respective alteration; green: detectable, yellow: the translocation is only detectable in conjunction with CBA, in which fluorescence in situ hybridization on metaphases identifies the partner chromosome of 14q11 (TRAD) or 7q34 (TRB); yellow shaded: basically detectable; however, commercially available FISH probes are lacking for translocations; for rare fusions a PCR has to be established, red: not detectable; CBA: chromosome banding analysis; FISH: fluorescence in situ hybridization, M: molecular genetics; WGS: whole genome sequencing. **B** The Sankey diagram shows the shift in classification depending on the method applied. The height of the bars represents the relative distribution of the genetic subgroups.

Based on results of CBA supplemented by FISH with probes for detection of rearrangements involving TRAD, TRB, *TLX1*, *TLX3, NUP98*, and *HOXA9/10* and RT-PCR for detection of *STIL*::*TAL1*, *SET*::*NUP214* and *PICALM*::*MLLT10* 131 T-ALL cases were assigned to the following nine genetically defined subgroups: TLX1: structural alterations involving *TLX1*: *n* = 22; TLX3: structural alterations involving *TLX3*: *n* = 10, TAL1: structural alterations involving *TAL1*: *n* = 3, HOXA9/10: structural alterations involving *HOXA9/10* genes: *n* = 4, SET::NUP214: *SET*::*NUP214* fusion: *n* = 7, MLLT10: fusions involving *MLLT10*: *n* = 4, NUP98: fusions involving *NUP98*: *n* = 3, MYB: structural alterations involving *MYB*: *n* = 2, rare fusions (*LEF1*-, *LMO2*- and *NKX2-3*-rearrangements): *n* = 4, and NOS: not otherwise specified - lacking all of the subgroup-defining alterations of groups 1–9: *n* = 71 (Fig. 1A, B).

The detection of abnormal T-ALL clones by CBA is hampered by reduced in vitro proliferation of leukemia cells leading to an insufficient number of metaphases or only metaphases with a normal karyotype from normal hematopoietic cells. Supplementary FISH or RT-PCR analyses were required for genetic subtype classification in 26/131 (20%) cases: 20 due to the cytogenetically cryptic nature of the abnormality and 6 due to insufficient in vitro proliferation of the T-ALL clone. Thus, in cases where no abnormalities have been detected, additional FISH screening should be performed. Furthermore, several abnormalities are not detectable by CBA due to its low resolution such as rearrangements of *BCL11B*::*TLX3*, *SET*::*NUP214* and *STIL*::*TAL1*. Therefore, for a comprehensive classification of T-ALL it is necessary to supplement CBA by FISH and RT-PCR.

Next, we evaluated whether WGS data can add relevant information for classification. Of note, all CBA ± FISH ± RT-PCR assignments were confirmed by WGS. In 13 cases, in which either no material for FISH was available (*n* = 4) or no FISH probes or RT-PCR were available for the detection of the respective abnormality (*n* = 9, *CCDC26*::*TLX3*, *DDX3X*::*MLLT10*, *XPO1*::*MLLT10*, TRB::*MYB*, TRB::*NOTCH1*; *MYB* enhancer mutation), WGS identified specific rearrangements (*TLX1*: 1; *TLX3*: 2, *HOXA9/10*: 2, *MLLT10*: 3, *MYB*: 2, rare rearrangements: 3 (TRB::*NOTCH1* (*n* = 1); *MYB* enhancer mutation (*n* = 2)). Further, 10 cases were assigned to the recently described *BCL11B*-rearranged subset (*CCDC26*::*BCL11B* (*n* = 2), *ARID1B*::*BCL11B* (*n* = 2), *SATB1*::*BCL11B* (*n* = 1), or *BCL11B* enhancer amplification (*n* = 5)) [4, 5]. Based on WGS data 83/131 cases (63%) were assigned to a specific genetic subgroup and only 37% of cases were labeled “not otherwise specified”. *BCL11B* rearrangements are found in T-ALL, MPAL and immature AML [4, 5], advocating the introduction of *BCL11B* FISH probes into routine diagnostics for classification of acute leukemia’s of ambiguous lineage according to the 5th edition of the WHO classification [1].

**Fig. 2:**
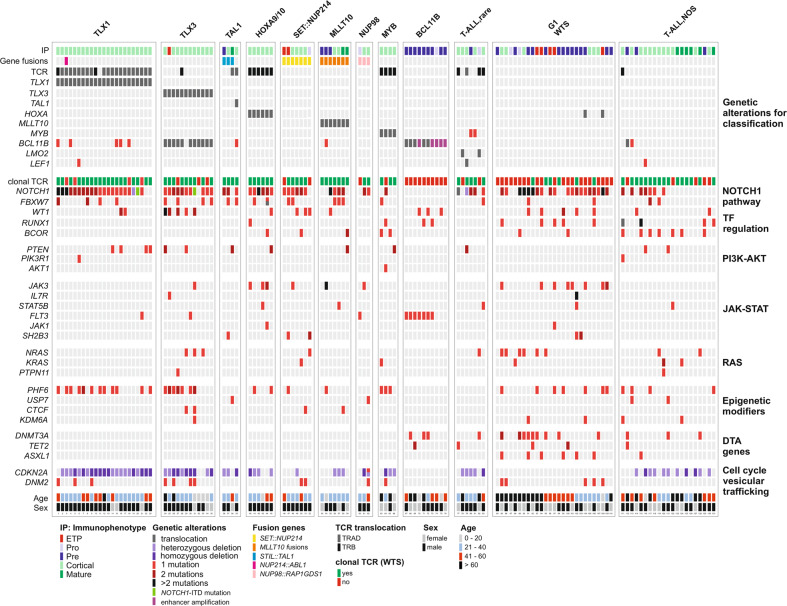
Profiling of T-ALL subtypes. Immunophenotype, gene fusions, translocations, deletions, mutations, the clonality status of TCR analyzed by WTS, age and sex are depicted. Mutated genes are labeled in red (one mutation), dark red (two mutations) or black (more than two mutations). Genetic alterations in *TLX1*, *TLX3*, *TAL1*, *HOXA9/10*, *MLLT10*, *NUP98*, *MYB*, *BCL11B*, and the presence of *SET::NUP214* fusion gene allows classification into 9 distinct subgroups. Cases with rare but recurrent changes are listed as T-ALL,rare (*LMO2*-Rearrangement, *n* = 2; TRAD*::LEF1*-Rearrangement, *n* = 1; TRB*::NOTCH1*-Rearrangement, *n* = 1; TRB*::NKX2-3*-Rearrangement, *n* = 1, Mutation in MYB Enhancer, *n* = 2). 27 cases lacking such genetic features have a similar gene expression profile (G1). The remaining cases with no apparent common features are classified as T-ALL, NOS. Each column of the plot represents an individual case. Genetic alterations are sorted according to classification and biological pathways.

A comprehensive clinical and genetic workup revealed that the subtypes showed distinct characteristics (Fig. 2). The TLX1- and TLX3 group were associated with young age (median age: 37 years, range 20–60 years; 22 years, 5–75 years), a strong male preponderance (male: female 6.7:1; 3:1), as well as a high rate of *CDKN2A* deletions (22/23, 96%; 10/12, 83%), a high frequency of *NOTCH1* (21/23, 91%; 11/12, 92%) and *PHF6* mutations (12/23,52%; 7/12, 58%). A high frequency of *WT1* mutations was found in the TLX3-group (5/12; 41.7%).

Patients assigned to the groups TAL1, HOXA9/10, SET::NUP214, MLLT10-fusions, and NUP98-fusions were also younger compared to the BCL11B- and NOS group (median age: 32.7, 30.6, 26.4, 28.9, and 32.3 vs 43.1, and 56.2 years) (Supplementary Table 1).

In line with published data, we found that the BCL11B group was characterized by the absence of *NOTCH1* mutation, *PHF6* mutations and *CDKN2A* deletion, and a high frequency of *FLT3* mutations (7/10 cases, 70%, ITD: *n* = 4; TKD: *n* = 3). While cases in the BCL11B group showed a high expression of *KIT* and *LMO2* (Supplementary Fig. 1), we found low *RAG1* and *RAG2* expression and the absence of TCR rearrangements (Supplementary Fig. 1, Fig. 2), supporting the hypothesis that the cell of origin is a primitive hematopoietic progenitor cell, in which the ectopic *BCL11B* expression induces a T-lineage transcriptional program. Cell type enrichment analyses [6] revealed that in the BCL11B-group granulocyte/macrophage progenitor and hematopoietic stem cells were more frequent than in the TLX1-, TLX3-, TAL1-group, in which dendritic cells, Th1 and Th2 cells were more frequent (Supplementary Fig. 2).

WTS has proven to be a valuable method for identification of new biological subtypes, e.g. in BCP-ALL [4, 7, 8]. Recently, a comprehensive analysis of 707 T-ALL transcriptome profiles identified 10 distinct subtypes (G1-G10) characterized by known and novel genetic aberrations and expression patterns [9]. Subgroups with high expression of *LYL1*/*LMO2* (G1), *GATA3* mutations (G2), *SPI1*-fusions (G3), *KMT2A*-rearrangements (G4), *MLLT10*-rearrangements (G5) and *HOXA10*-fusions (G6) might represent the early T-cell progenitor, pro/precortical/cortical stage with a relatively high age of disease onset. Lymphoblasts with high expression of *TLX3* (G7) and *TLX1* (G8) could be blocked at the cortical/postcortical stage, whereas those with high expression of *NKX2-1* (G9) or *TAL1*/*LMO1* (G10) might correspond to cortical/postcortical/mature stages of T-cell development. We stratified our cohort into the G1-G10 expression groups (Supplementary Table 1, Supplementary Fig. 3). Subgroups G2/3/4/9 were not detectable in our cohort, as subgroups G3/4/9 are mainly present in childhood T-ALL and the G2 subgroup seems to be very rare [9]. However, the majority of our cases assigned to the NOS group belonged to the G1 group (27/48 cases, 60%). Within this G1/NOS group a subset of 21 cases did not harbor a clonal TCR rearrangement, showed low expression of *RAG1* and *RAG2* (Fig. 2, Supplementary Fig. 1), a high frequency of *DNMT3A* (7/21; 33%) and *ASXL1* mutations (4/21; 19%), no *CDKN2A* deletions and a higher median age (58 years), thus, characteristics shared with the BCL11B group. Mutations in genes involved in DNA methylation (e.g. *DNMT3A* and *TET2*) have been associated with impaired differentiation of hematopoietic stem cells [10, 11]. In our cohort, mutations in *DNMT3A, TET2* and *ASXL1* were exclusively detected in patients assigned to the BCL11B-, G1- or NOS group. Gene set enrichment analyses identified a strong, significant correlation of *DNMT3A* mutations with increased age (Supplementary Fig. 4). In contrast to the BCL11B group, *NOTCH1* (16/21; 76%) and *PHF6* (8/21; 38%) mutations were frequent in the G1- and NOS-groups.

Additionally, we identified seven cases with a distinct gene expression pattern characterized by high expression of *KCNG3*, *PTPRK*, and *SCRN1* as well as low expression of *ERG*, *HOXA10*, *P2RY1*, *TTC28*, *ZBTB8A*, and *ZNF618* (Supplementary Fig. 5). All cases were classified as cortical T-ALL and harbored a clonal TCR rearrangement. In 5 cases a translocation involving TRB and *MYB* (*n* = 3), *RUNX1* (*n* = 1) and *NOTCH1* (*n* = 1) was observed. Interestingly, 5/7 cases harbored *BCOR* and *PHF6* co-mutations, which was observed in only two other cases in the entire cohort.

Although the cohort size is quite small we performed explorative overall survival (OS) analysis (Supplementary Fig. 6). The median survival of the total cohort was not reached with 63.4% surviving five years. The TLX1 and HOXA group demonstrated a significantly more favorable outcome, especially compared to MYB, T-ALL,NOS, or T-ALL,rare (Supplementary Table 2).

In conclusion, CBA supplemented by a FISH panel comprising six probe sets and RT-PCR screening for *STIL*::*TAL1*, *PICALM*::*MLLT10*, and *SET*::*NUP214* allows to classify 46% of T-ALL into distinct genetically defined entities. WGS can help to further refine T-ALL classification and assign an additional 17% to distinct genetic subgroups. Due to the fact, that gene expression analysis is not a standard diagnostic technique yet we believe that a first step towards a genetic classification into a routine setting should be based on broadly available techniques. In a second step unclassified cases can be resolved by novel methods. In addition to primary genetic events used for classification, secondary events are prognostically relevant and are used for stratifying patients in clinical trials. Thus, we support a biomarker-driven classification also in T-ALL to allow subtype-associated treatment, compare responses and lead to comparability between trials as an essential step towards personalized medicine.

## Supplementary information

Supplementary Material

Supplementary Figures

Supplementary Figure 1

Supplementary Figure 2

Supplementary Figure 3

Supplementary Figure 4

Supplementary Figure 5

Supplementary Figure 6

Supplementary Tables

## Data Availability

The datasets generated during and/or analyzed during the current study are available from the corresponding author on reasonable request.
