# Correction: How T-lymphoblastic leukemia can be classified based on genetics using standard diagnostic techniques enhanced by whole genome sequencing

**DOI:** 10.1038/s41375-022-01790-z

**Published:** 2022-12-16

**Authors:** Janine Müller, Wencke Walter, Claudia Haferlach, Heiko Müller, Irene Fuhrmann, Martha-Lena Müller, Henning Ruge, Manja Meggendorfer, Wolfgang Kern, Torsten Haferlach, Anna Stengel

**Affiliations:** MLL Munich Leukemia Laboratory, Max-Lebsche-Platz 31, 81377 Munich, Germany

**Keywords:** Acute lymphocytic leukaemia, Cancer genetics

Correction to: *Leukemia* 10.1038/s41375-022-01743-6, published online 5 November 2022

The article “How T-lymphoblastic leukemia can be classified based on genetics using standard diagnostic techniques enhanced by whole genome sequencing”, written by Janine Müller, Wencke Walter, Claudia Haferlach, Heiko Müller, Irene Fuhrmann, Martha-Lena Müller, Henning Ruge, Manja Meggendorfer, Wolfgang Kern, Torsten Haferlach and Anna Stengel, was originally published electronically on the publisher’s internet portal on 5 November 2022 without open access. With the author(s)’ decision to opt for Open Choice the copyright of the article changed on 15 November 2022 to © The Author(s) 2022 and the article is forthwith distributed under a Creative Commons Attribution 4.0 International License, which permits use, sharing, adaptation, distribution and reproduction in any medium or format, as long as you give appropriate credit to the original author(s) and the source, provide a link to the Creative Commons licence, and indicate if changes were made. The images or other third party material in this article are included in the article’s Creative Commons licence, unless indicated otherwise in a credit line to the material. If material is not included in the article’s Creative Commons licence and your intended use is not permitted by statutory regulation or exceeds the permitted use, you will need to obtain permission directly from the copyright holder. To view a copy of this licence, visit http://creativecommons.org/licenses/by/4.0/.

