# Supplementary material for: How T-lymphoblastic leukemia can be classified based on genetics using standard diagnostic techniques enhanced by whole genome sequencing
Source: Leukemia. 2022 Nov 5;37(1):217–21. doi: 10.1038/s41375-022-01743-6 (PMC9883150; doi:10.1038/s41375-022-01743-6)
Supplement: Supplementary file 1 — Supplementary Material [file 41375_2022_1743_MOESM1_ESM.docx]

**Supplementary Material**

**Material and Methods**

**Patients and samples**

For diagnostic work-up, 131 patients with newly diagnosed T-ALL (8 ETP-ALL, 6 pro-T-ALL, 31 pre-T-ALL, 77 cortical-T-ALL and 9 mature T-ALL) sent to the MLL Leukemia Laboratory between 05/2008–12/2020 were selected based on sample availability for WTS and WGS. The diagnosis was established following WHO guidelines. Immunophenotyping, cytomorphology, cytogenetics and molecular genetics were performed as previously published (1, 2). In addition, WGS and WTS was performed in all cases. The T-ALL cohort comprised 40 female (31%) and 91 male (69%) patients with a median age of 37.8 years (range 0.8 – 90.8 years) at diagnosis (Supplementary table 1). Follow-up data were available for **125** of 131 patients (median follow up: 43.1 months, range: 1 day – 159.6 months). Samples from either bone marrow (BM) and/or peripheral blood (PB) were collected. Of the 89 bone marrow samples, six samples had bone marrow infiltration of less than 25% blasts (range: 14-23%). 42 analyses were performed from peripheral blood and showed a blast population between 20% and 99%. All cases were prepared for immunophenotyping employing standard staining, measurement and analysis protocols (3-5), as well as genetic analyses. To that end, DNA and total RNA were extracted using the MagNA Pure 96 Instrument and the MagNAPure96 DNA and Viral NA LV Kit and MagNA Pure 96 Cellular RNA LV Kit, respectively (Roche LifeScience, Mannheim, Germany).

**Chromosome banding analysis**

Bone marrow or peripheral blood cells were cultivated unstimulated or stimulated with thymidine and/or interleukins (IL-2, IL-3, IL-6, IL-7) or TPA (Phorbol-12-myristate-13 acetate) for 24 and 48 hr. Chromosome banding analysis (CBA) was performed for all 131 cases as previously described according to standard methods (2). 15 to 30 metaphase cells were analyzed according to the International System for Human Cytogenetic Nomenclature (6, 7).

**FISH with locus-specific probes**

FISH on interphase nuclei or metaphase chromosomes was performed for screening or confirmation using commercially available probes for *TLX1* (10q24) (TLX1 break apart, CytoCell, Cambridge, United Kingdom), *TLX3* (5q35) (XL TLX3 break apart, MetaSystems, Altlussheim, Germany), *BCL11B::TLX3* (t(5;14)(q35;q32)) (BCL11B::TLX3 dual fusion, CytoCell, Cambridge, United Kingdom), *TRAD* (14q11) (XL TRA/D break apart, MetaSystems, Altlussheim, Germany), *TRB* (7q34) (TCRB break apart, CytoCell, Cambridge, United Kingdom), *HOXA9* (7p15) (CL HOXA9 break apart , MetaSystems, Altlussheim, Germany) and *NUP98* (11p15) (XL NUP98 break apart, MetaSystems, Altlussheim, Germany), according to the protocol of the manufacturer. The probes were analyzed using a Zeiss AxioImager microscope (Zeiss, Jena, Germany) or the analyzing system ISIS (MetaSystems, Altlussheim, Germany).

**Whole-chromosome-painting and 24-color FISH (MFISH)**

Aberrant karyotypes were analyzed with commercially available probes for whole-chromosome-painting (MetaSystems, Altlussheim, Germany) or MFISH (24XCyte Human Multicolor FISH probe, MetaSystems, Altlussheim, Germany), according to the protocol of the manufacturer. Analysis was done on an AxioImager with filters for DAPI, FITC, Texas Red, Cy3, Cy5, and DEAC (Feuerbacher,Tübingen,Germany) using ISIS software (MetaSystems, Altlussheim, Germany).

**Whole-Genome Sequencing**

WGS was applied with a median coverage of 90x. Library preparation was performed using TruSeq DNA PCR-Free HT sample preparation kit (Illumina, San Diego, CA, USA) according to the manufacturer’s protocol. 150bp paired-end reads where generated on Illumina HiSeqX and NovaSeq 6000 machines. Read mapping (Isaac aligner iSAAC-03.16.02.19, Ensembl GRCh37) and tumor/normal variant calling were performed using Illumina’s WGS app version 5.0 and tumor normal app 3.0 for analysis of genomic data. Matched normal samples were not available, so a mixture of genomic DNA from bone marrow samples of multiple anonymous donors was used as normal control.

**Whole-Transcriptome Sequencing**

Stranded RNA libraries were constructed from ribosomal RNA-depleted RNA using TruSeq Total Stranded RNA kit (Illumina, San Diego, CA, USA). 2x101bp paired-end reads were analyzed on the NovaSeq 6000 system with a median of 50 mio reads per sample. FASTQ files were generated applying Illumina’s bcl2fastq software (v1.8.4) and further preprocessed using BaseSpace’s RNA-seq Alignment app (v2.0.1). Applying default settings reads were mapped with STAR aligner (v2.5.0a) to the human reference genome hg19 (RefSeq annotation), gene counts were determined (Cufflinks, v2.2.1) and Manta (v0.28.0) (8), Arriba (9) and STAR-Fusion (10) were used for fusion calling. The resulting estimated gene counts were pre-processed and normalized, applying Trimmed mean of M-values normalization method (11). Normalized counts per million (CPM) were used as a proxy of gene expression in each sample.

**Mutational analysis and variant filtering**

For mutational analysis, data on the whole exome was used. To exclude artifacts, variants with a VAF of <0.15 were either confirmed via paired-end targeted sequencing on Illumina MiSeq devices or excluded from further analysis, if no primers for the region of interest were available. Additionally, only variants in regions described as a high confidence by the Genome in a Bottle Consortium (12) were analyzed further. Moreover, genes prone for generating artefacts by their location in problematic genomic regions based on Fuentes Fajardo et al. (13) were excluded from further analysis.

To remove potential germline variants, each variant was queried against the gnomAD database (14) and variants with global population frequencies >1% where excluded. Moreover, variants were eliminated if a germline origin was suggested by ClinVar database (15) and simultaneously a VAF ranging from 0.45 to 0.55 was detected.

Additionally, only mutations with an MLL predictor (16) score of >0.5 were used for this cohort. The MLL predictor is a classifier for potential pathogenicity of missense variants in hematological malignancies. Based on multiple *in silico* pathogenicity prediction algorithms and phylogenetic conservation scores available in the dbNSFP database (17), a random forest model was trained (implemented in R, using the randomForest library) with a training set consisting of missense variants, which were observed ≥10 times in routine diagnostics at the MLL. Classification accuracy is estimated to be 96.8% by 10-fold cross-validation and out-of-bag estimation of error rates. MLL predictor scores range from 0 (benign) to 1 (pathogenic). Furthermore, the genomic region of the detected mutations was queried against the Ensembl GRCh37 (18) and the Global Alliance for Genomics and Health (GA4GH) database (19), and regions classified as decoy, as well as regions located in segmental duplication regions in combination with low map complexity, were excluded from further analysis. Final analysis was performed only on protein-altering and splice-site variants as annotated by the Illumina annotation engine.

**Gene enrichment analysis**

Gene enrichment analysis (20) was performed using GSEA v2.2.1. software (<https://software.broadinstitute.org/cancer/software/gsea/wiki/index.php/GSEA_v2.2.x_Release_Notes>). Analysis was run on pre-ranked lists using 1000 permutations and weighted enrichment statistic.

**Cell type enrichment analysis**

### Cellular heterogeneity of the different samples was assessed with xCell (21). The enrichment scores were normalized and subsequently visualized with the R package ggplot2 (ggplot2_3.3.6).

### T-cell receptor rearrangement analysis

### Fastq reads extracted from RNASeq bam files were aligned to reference V, D, J, and C genes of T-cell receptors (TCR) and assembled into clonotypes using MiXCR software (22). TCR reference sequences used were included in the MiXCR software as repseqio.v1.8.json (TRA/TRD, NG_001332.2; TRB, NG_001333.2; TRG, NG_001336.2) (22). TCR rearrangements with (i) cloneCount < 5 and (ii) cloneFraction < 0.05 were excluded.

### Statistical analysis

### Statistical analyses were performed using SPSS software, version 19.0.0 (IBM Corporation, Armonk, NY, USA), Prism software, version 8.0 (GraphPad Software, Inc., La Jolla, CA, USA), R, version 3.5.1 software (R Foundation for Statistical Computing, Vienna, Austria) and routines from the biostatistics software repository Bioconductor. For correlation analysis, the Pearson correlation coefficient (bivariate correlation) was used. For comparison of median values, the independent samples t-test was applied. For comparison of overall survival, the log-rank test was used (SPSS).

**References**

1. Kern W, Voskova D, Schoch C, Hiddemann W, Schnittger S, Haferlach T. Determination of relapse risk based on assessment of minimal residual disease during complete remission by multiparameter flow cytometry in unselected patients with acute myeloid leukemia. Blood. 2004;104(10):3078-85.

2. Schoch C, Schnittger S, Bursch S, Gerstner D, Hochhaus A, Berger U, et al. Comparison of chromosome banding analysis, interphase- and hypermetaphase-FISH, qualitative and quantitative PCR for diagnosis and for follow-up in chronic myeloid leukemia: a study on 350 cases. Leukemia. 2002;16(1):53-9.

3. Bene MC, Castoldi G, Knapp W, Ludwig WD, Matutes E, Orfao A, et al. Proposals for the immunological classification of acute leukemias. European Group for the Immunological Characterization of Leukemias (EGIL). Leukemia. 1995;9(10):1783-6.

4. Kern W, Bacher U, Haferlach C, Alpermann T, Dicker F, Schnittger S, et al. Frequency and prognostic impact of the aberrant CD8 expression in 5,523 patients with chronic lymphocytic leukemia. Cytometry B Clin Cytom. 2012;82(3):145-50.

5. Porwit-MacDonald A, Bjorklund E, Lucio P, van Lochem EG, Mazur J, Parreira A, et al. BIOMED-1 concerted action report: flow cytometric characterization of CD7+ cell subsets in normal bone marrow as a basis for the diagnosis and follow-up of T cell acute lymphoblastic leukemia (T-ALL). Leukemia. 2000;14(5):816-25.

6. McGowan-Jordan J, Simons A, Schimd M. ISCN : an international system for human cytogenomic nomenclature (2016). Basel ; New York : Karger. 2016.

7. McGowan-Jordan J, Hastings R, Moore S. ISCN 2020: An International System for Human Cytogenomic Nomenclature. Basel: Karger. 2020.

8. Chen X, Schulz-Trieglaff O, Shaw R, Barnes B, Schlesinger F, Kallberg M, et al. Manta: rapid detection of structural variants and indels for germline and cancer sequencing applications. Bioinformatics. 2016;32(8):1220-2.

9. Uhrig S, Ellermann J, Walther T, Burkhardt P, Frohlich M, Hutter B, et al. Accurate and efficient detection of gene fusions from RNA sequencing data. Genome Res. 2021;31(3):448-60.

10. Haas BJ, Dobin A, Stransky N, Li B, Yang X, Tickle T, et al. STAR-Fusion: Fast and Accurate Fusion Transcript Detection from RNA-Seq. bioRxiv. 2017:120295.

11. Robinson MD, Oshlack A. A scaling normalization method for differential expression analysis of RNA-seq data. Genome Biol. 2010;11(3):R25.

12. Zook JM, Catoe D, McDaniel J, Vang L, Spies N, Sidow A, et al. Extensive sequencing of seven human genomes to characterize benchmark reference materials. Sci Data. 2016;3:160025.

13. Fuentes Fajardo KV, Adams D, Program NCS, Mason CE, Sincan M, Tifft C, et al. Detecting false-positive signals in exome sequencing. Hum Mutat. 2012;33(4):609-13.

14. Karczewski KJ, Francioli LC, Tiao G, Cummings BB, Alfoldi J, Wang Q, et al. The mutational constraint spectrum quantified from variation in 141,456 humans. Nature. 2020;581(7809):434-43.

15. Landrum MJ, Lee JM, Benson M, Brown GR, Chao C, Chitipiralla S, et al. ClinVar: improving access to variant interpretations and supporting evidence. Nucleic Acids Res. 2018;46(D1):D1062-D7.

16. Hutter S, Baer C, Walter W, Kern W, Haferlach C, Haferlach T. A Novel Machine Learning Based in silico Pathogenicity Predictor for Missense Variants in a Hematological Setting. Blood. 2019;134(Supplement_1):2090.

17. Liu X, Wu C, Li C, Boerwinkle E. dbNSFP v3.0: A One-Stop Database of Functional Predictions and Annotations for Human Nonsynonymous and Splice-Site SNVs. Hum Mutat. 2016;37(3):235-41.

18. Yates AD, Achuthan P, Akanni W, Allen J, Allen J, Alvarez-Jarreta J, et al. Ensembl 2020. Nucleic Acids Res. 2020;48(D1):D682-D8.

19. Terry SF. The global alliance for genomics & health. Genet Test Mol Biomarkers. 2014;18(6):375-6.

20. Subramanian A, Tamayo P, Mootha VK, Mukherjee S, Ebert BL, Gillette MA, et al. Gene set enrichment analysis: a knowledge-based approach for interpreting genome-wide expression profiles. Proc Natl Acad Sci U S A. 2005;102(43):15545-50.

21. Aran D, Hu Z, Butte AJ. xCell: digitally portraying the tissue cellular heterogeneity landscape. Genome Biology. 2017;18(1):220.

22. Bolotin DA, Poslavsky S, Mitrophanov I, Shugay M, Mamedov IZ, Putintseva EV, et al. MiXCR: software for comprehensive adaptive immunity profiling. Nat Methods. 2015;12(5):380-1.
