# Supplementary material for: How T-lymphoblastic leukemia can be classified based on genetics using standard diagnostic techniques enhanced by whole genome sequencing
Source: Leukemia. 2022 Nov 5;37(1):217–21. doi: 10.1038/s41375-022-01743-6 (PMC9883150; doi:10.1038/s41375-022-01743-6)
Supplement: Supplementary file 2 — Supplementary Figures [file 41375_2022_1743_MOESM2_ESM.docx]

**Supplementary figure 1. Gene expression of *LMO2*, *KIT*, *RAG1* and *RAG2*.** The BCL11B subgroup shows a significant overexpression of *KIT* and *LMO2* and a low expression of *RAG1* and *RAG2*.

**Supplementary figure 2. Comparison of cell-type enrichment scores.** In the BCL11B-group granulocyte/macrophage progenitor and hematopoietic stem cells were more frequent than in the TLX1-, TLX3-, TAL1-group in which dendritic cells, Th1 and Th2 cells were more frequent.

**Supplementary figure 3. T-ALL subtypes by gene expression.** Gene expression profiling (GEP) of 131 cases shown in an uniform manifold approximation and projection (UMAP) plot. T-ALL subtypes highlighted in different colors. Each symbol visualizes a sample; triangle: clonal T-cell receptor rearrangement, dot: no clonal T-cell receptor rearrangement

**Supplementary figure 4. Gene set enrichment analyses.** *DNMT3A* mutations are preferentially found in older patients. Ranked list was generated by sorting samples by decreasing age at first diagnosis. The „gene“ set comprises samples with mutated *DNMT3A*. Enrichment Score (ES) 16, Normalized Enrichment Score (NES) 0.72, FDR q-value 0.0.

**Supplementary figure 5. Distinct gene expression pattern of 7 cases within the NOS group.** Within the NOS group, 7 cases (outlier) showed a distinct expression pattern with overexpression of *KCNG3*, *PTPRK*, and *SCRN1* as well as a low expression of *ERG*, *HOXA10*, *P2RY1*, *TTC28*, *ZBTB8A*, and *ZNF618.*

**Supplementary figure 6. Overall survival according to genetic subgroups.** Kaplan-Meier plot depicting the OS within the specific molecular subgroups. The TLX1 and HOXA group demonstrated a significantly more favorable outcome, especially compared to MYB, T-ALL,NOS, or T-ALL,rare (TLX1 vs. MYB, p=0.001; TLX1 vs. T-ALL,rare, p=0.001; TLX1 vs. T-ALL,NOS, p=0.006; HOXA vs. MYB, p=0.029; HOXA vs. T-ALL,rare, p=0.012). Median OS for specific subgroups: BCL11B (35 months), MYB (5 months), T-ALL,rare (13 months), T-ALL,NOS (25 months); not reached for groups: HOXA9/10, MLLT10, NUP98, *SET::NUP214*, TAL1, TLX1, TLX3.

**Supplementary Table 1.** Summary of all data regarding gender, age, molecular subgroup, entity-defining cytogenetic, fusion or rearrangements, clonal TCR status, mutations, karyotype and FISH formula according to ISCN.

**Supplementary Table 2.** Summary of overall survival (OS) data analyzed in patients in the respective subgroups. Only significant p-values are given (pairwise comparison between subgroups). n.s.: not significant; n.r.: not reached

| **Group** | **Median OS** | **vs. BCL11B** | **vs. HOXA9/10** | **vs. MLLT10** | **vs. MYB** | **vs. NUP98** | **vs. T-ALL,rare** | **vs. *SET::NUP214*** | **vs. T-ALL-NOS** | **vs. Tal1** | **vs. TLX1** | **vs. TLX3** |
| --- | --- | --- | --- | --- | --- | --- | --- | --- | --- | --- | --- | --- |
| **BCL11B**  n=9 | 35 | - | n.s. | n.s. | n.s. | n.s. | n.s. | n.s. | n.s. | n.s. | n.s. | n.s. |
| **HOXA9/10**  n=6 | n.r. | n.s. | - | n.s. | 0.03 | n.s. | 0.01 | n.s. | n.s. | n.s. | n.s. | n.s. |
| **MLLT10**  n=7 | n.r. | n.s. | n.s. | - | n.s. | n.s. | n.s. | n.s. | n.s. | n.s. | n.s. | n.s. |
| **MYB**  n=4 | 5 | n.s. | 0.03 | n.s. | - | 0.05 | n.s. | n.s. | n.s. | n.s. | 0.001 | n.s. |
| **NUP98**  n=3 | n.r. | n.s. | n.s. | n.s. | 0.05 | - | n.s. | .s. | n.s. | n.s. | n.s. | n.s. |
| **T-ALL,rare**  n=7 | 13 | n.s. | 0.01 | n.s. | n.s. | n.s. | - | n.s. | n.s. | n.s. | 0.001 | 0.05 |
| ***SET::NUP214***  n=7 | n.r. | n.s. | n.s. | n.s. | n.s. | n.s. | n.s. | - | n.s. | n.s. | n.s. | n.s. |
| **T-ALL,NOS**  n=46 | 25 | n.s. | n.s. | n.s. | n.s. | n.s. | n.s. | n.s. | - | n.s. | 0.01 | n.s. |
| **TAL1**  n=4 | n.r. | n.s. | n.s. | n.s. | n.s. | n.s. | n.s. | n.s. | n.s. | - | n.s. | n.s. |
| **TLX1**  n=21 | n.r. | n.s. | n.s. | n.s. | 0.001 | n.s. | 0.001 | n.s. | 0.01 | n.s. | - | n.s. |
| **TLX3**  n=11 | n.r. | n.s. | n.s. | .s. | n.s. | n.s. | 0.05 | n.s. | n.s. | n.s. | n.s. | - |
