# Supplementary figures and images for: How T-lymphoblastic leukemia can be classified based on genetics using standard diagnostic techniques enhanced by whole genome sequencing
Source: Leukemia. 2022 Nov 5;37(1):217–21. doi: 10.1038/s41375-022-01743-6 (PMC9883150; doi:10.1038/s41375-022-01743-6)

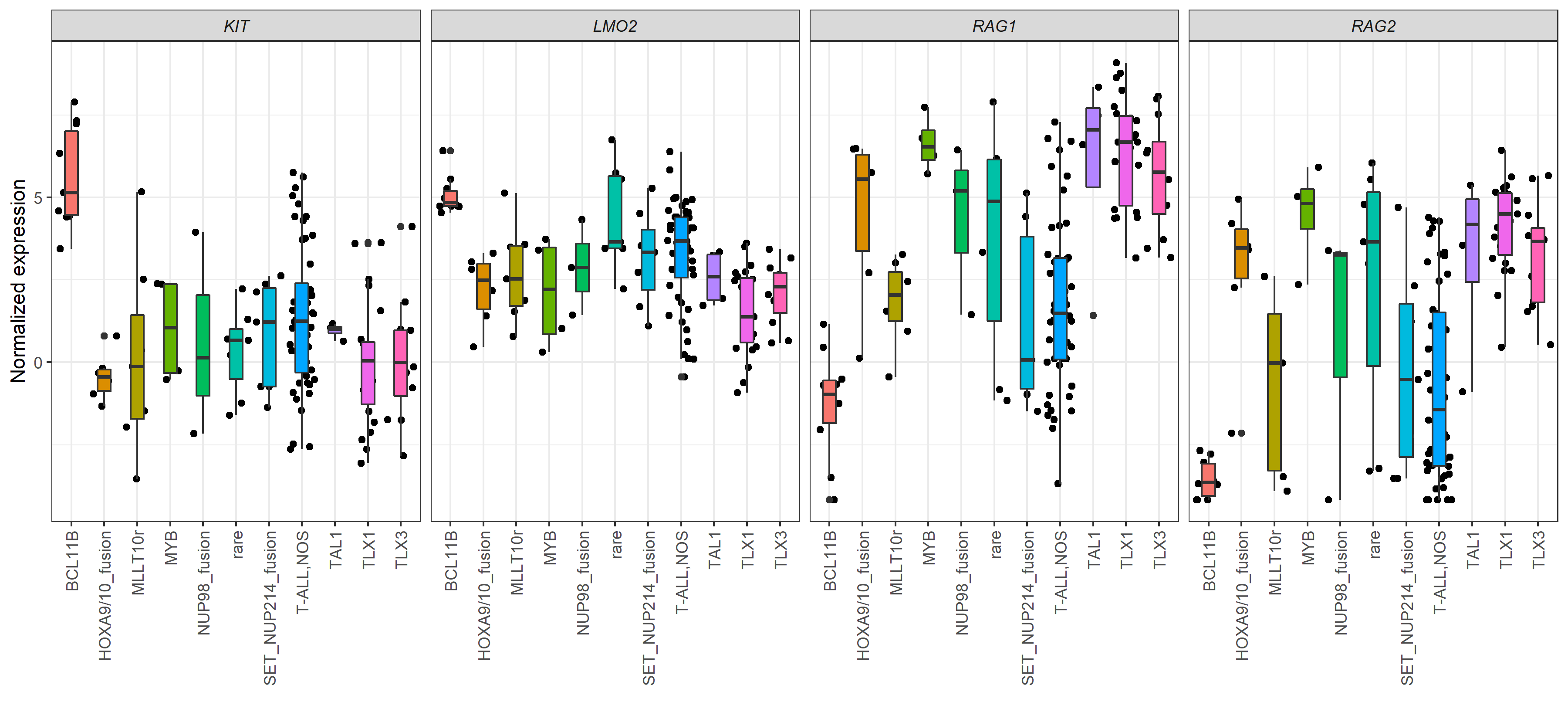

Supplement: Supplementary file 3 — Supplementary Figure 1 [file 41375_2022_1743_MOESM3_ESM.png]

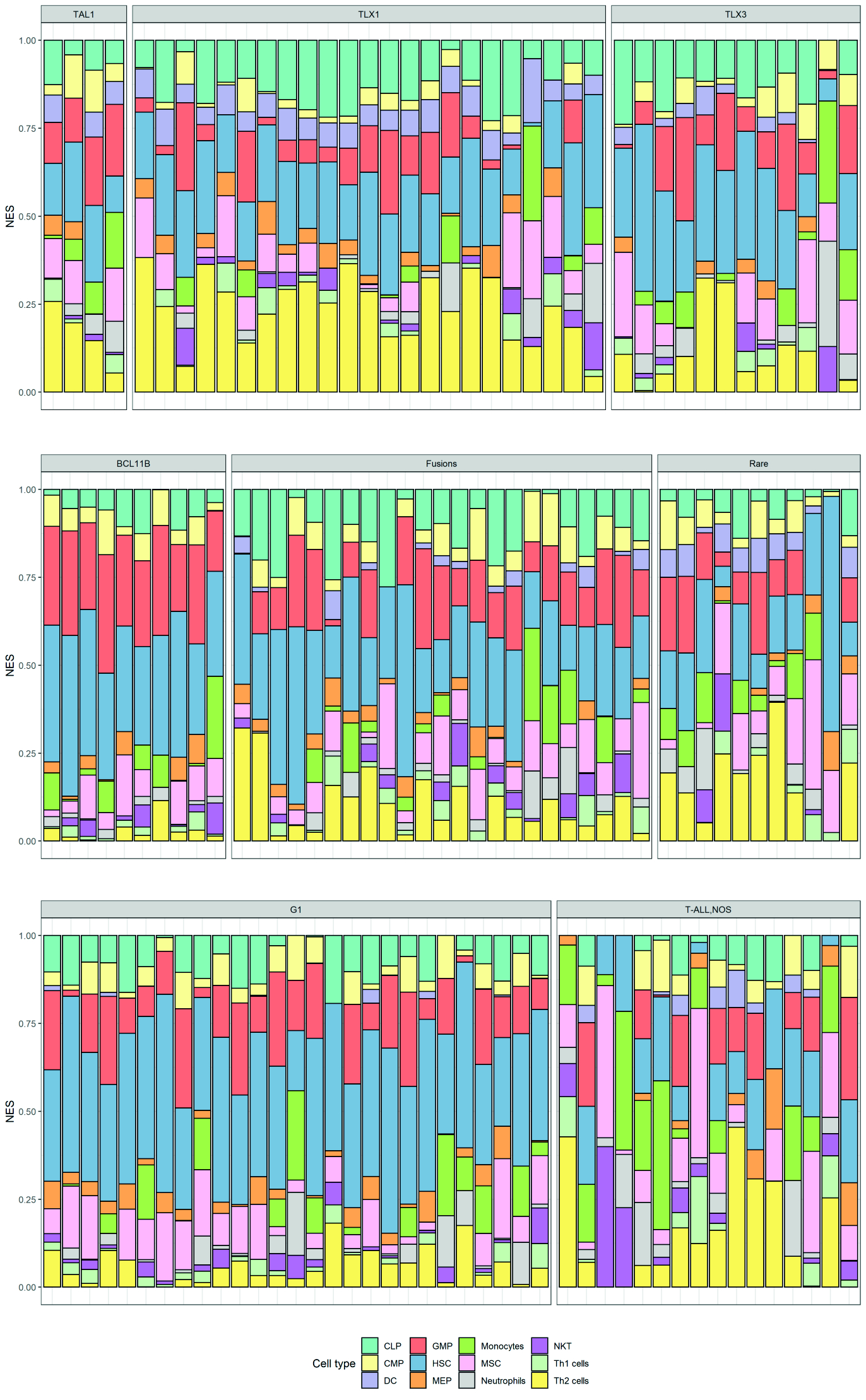

Supplement: Supplementary file 4 — Supplementary Figure 2 [file 41375_2022_1743_MOESM4_ESM.jpg]

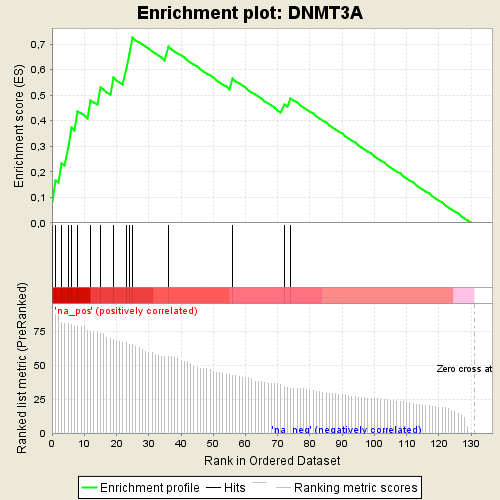

Supplement: Supplementary file 6 — Supplementary Figure 4 [file 41375_2022_1743_MOESM6_ESM.png]

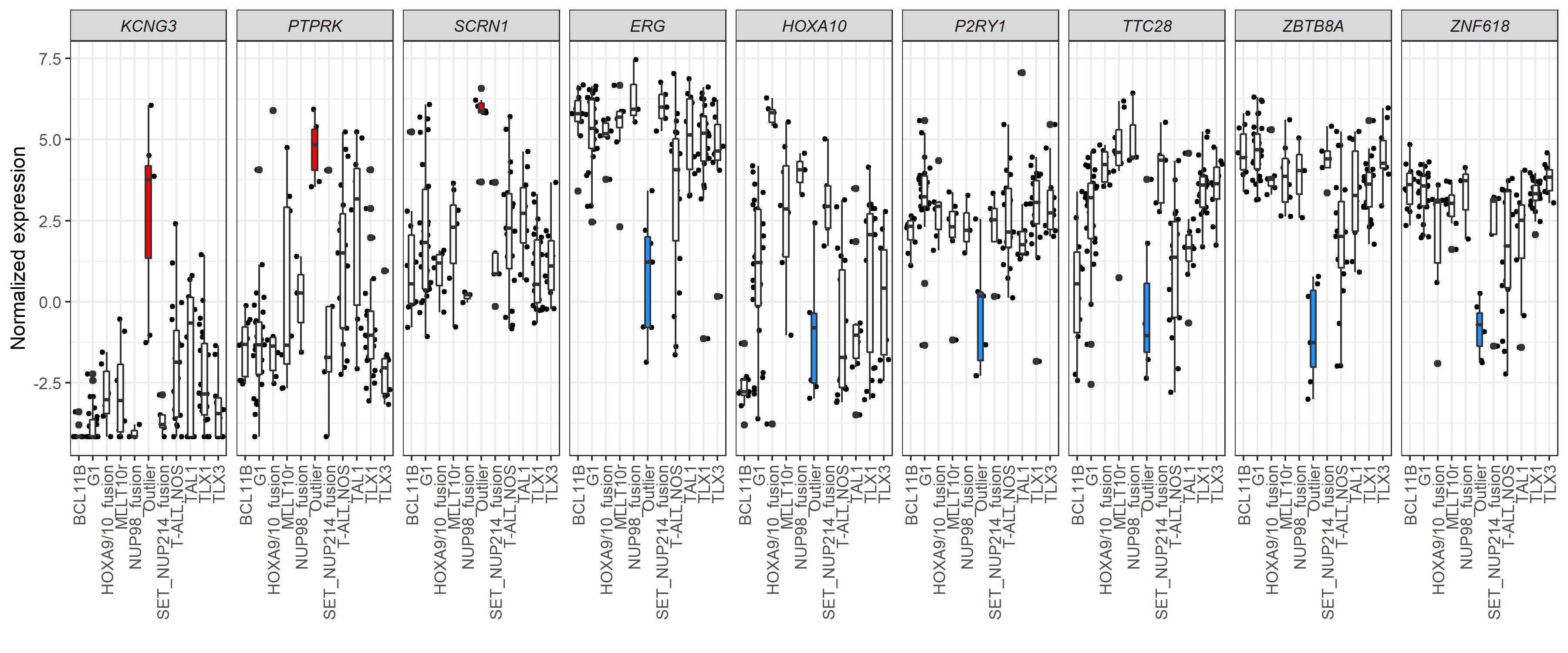

Supplement: Supplementary file 7 — Supplementary Figure 5 [file 41375_2022_1743_MOESM7_ESM.png]

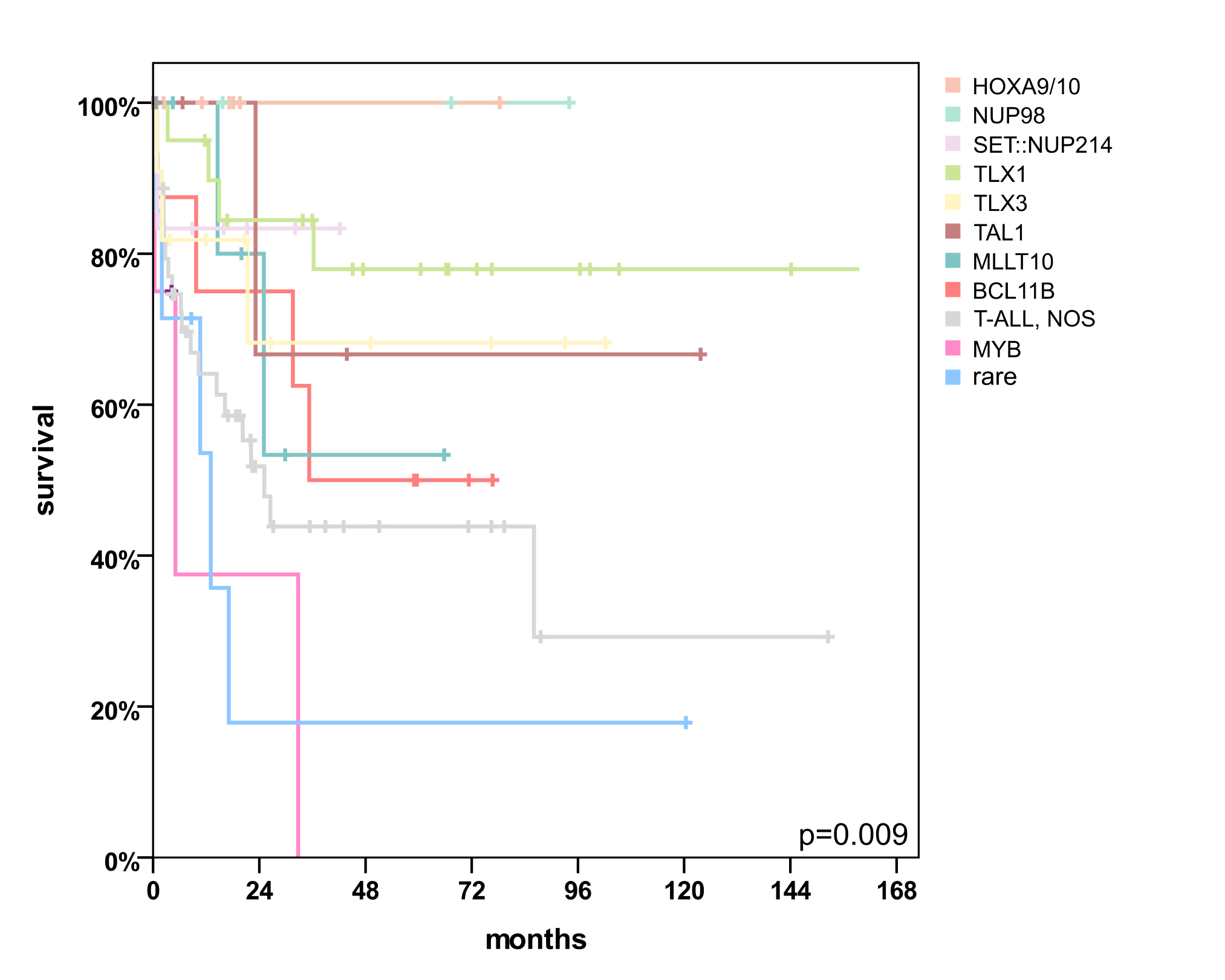

Supplement: Supplementary file 8 — Supplementary Figure 6 [file 41375_2022_1743_MOESM8_ESM.png]
